# Anterior capsule phimosis and capsular block syndrome in a patient with Steinert myotonic dystrophy: a case report

**DOI:** 10.1186/1757-1626-2-9298

**Published:** 2009-12-10

**Authors:** Nicola Rosa, Michele Lanza, Maddalena De Bernardo, Maria Borrelli, Luisa Politano

**Affiliations:** 1 Centro Grandi Apparecchiature, 2nd University of Naples, Naples, Italy; 2 Eye Department, 2nd University of Naples, Naples, Italy; 3 Department of Experimental Medicine, Cardiomyology and Medical Genetics 2nd University of Naples, Naples, Italy

## Abstract

A 55-year-old man with myotonic dystrophy underwent phacoemulsification with IOL implantation in the right eye.

3 months after surgery, the patient showed a decreased visual acuity and an intraocular pressure (IOP) of 30 mmHg. Slit lamp examination showed a dense fibrosis of the anterior capsule with capsulorexis' shrinkage. Gonioscopy showed a closed angle. After a YAG laser iridotomy no decrease in the IOP was detected; following surgical peeling of the anterior capsule, the slit lamp showed a distended capsular bag. A YAG laser posterior capsulotomy was performed, without decrease in the IOP. Myotonic patients need to be closely followed up after cataract surgery, because in case of CBS development a prompt posterior capsulotomy could avoid more severe complications.

## Background

Cataract is one of the most common eye disease in patients with Steinert myotonic dystrophy.

Anterior capsule phimosis (ACP) is one of the most common complications after continuous curvilinear capsulorexis (CCC) [1].

The degree of ACP is believed to be related to some types of disease such as pseudoexfoliation syndrome [2], diabetic retinopathy [3], retinitis pigmentosa [4], myotonic dystrophy [5].

Another rare complication of CCC is the capsular block syndrome (CBS) that is characterized by distension and accumulation of a liquefied substance inside the capsular bag.

We report a case of CBS and ACP in a patient with Steinert myotonic dystrophy.

## Case Report

A 55-year-old man with Steinert myotonic dystrophy was referred to us for cataract surgery. His best spectacle corrected visual acuity (BSCVA) was hand motion in the right eye (RE) and 20/200 in the left eye (LE). The intraocular pressure (IOP) was 12 mmHg in the RE and 10 mmHg in the LE. Slit lamp examination showed a total cataract in the RE and a dense corticonuclear opacity in the LE. The patient underwent phacoemulsification in the RE through a 3.00 mm superior corneal incision under peribulbar anesthesia with capsular staining with methilen blue. A 21 diopters single-piece hydrophilic acrylic IOL (Akreos Adapt, Bausch & Lomb) was implanted in the capsular bag after a 5.0 mm CCC was created. One month after surgery his BSCVA was 20/25 with sph +1.0. At this time he underwent phacoemulsification in his LE with a 23.5 diopter single-piece hydrophobic acrylic foldable lens (Hoya) implanted with the same modalities of the RE. 15 days later BSCVA was 20/20 with sph -0.50 D.

Two months after the second surgery the clinical picture in the LE was unchanged, but the RE showed a BSCVA of 20/50 with sph. -0.50 D with a myopic shift and an IOP of 30 mmHg.

**Figure 1:**
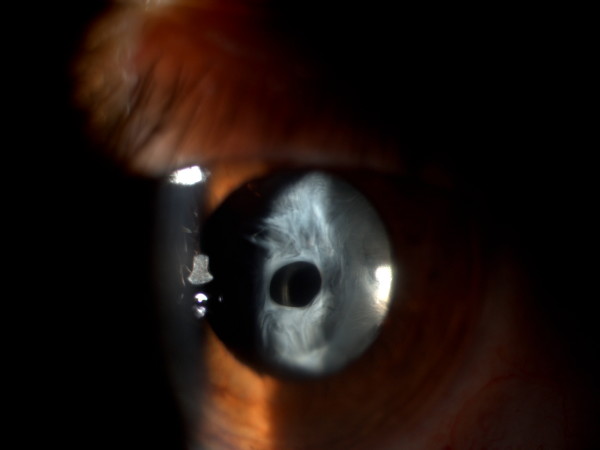
**Slit lamp examination showing a dense fibrosis of the anterior capsule with anterior capsule phimosis**.

Slit lamp examination showed a dense fibrosis of the anterior capsule with ACP (Fig. 1) that made difficult to visualize the posterior structures of the eye.

Gonioscopy showed a closed angle, and a YAG laser iridotomy was performed, but no decrease in the IOP was achieved.

**Figure 2:**
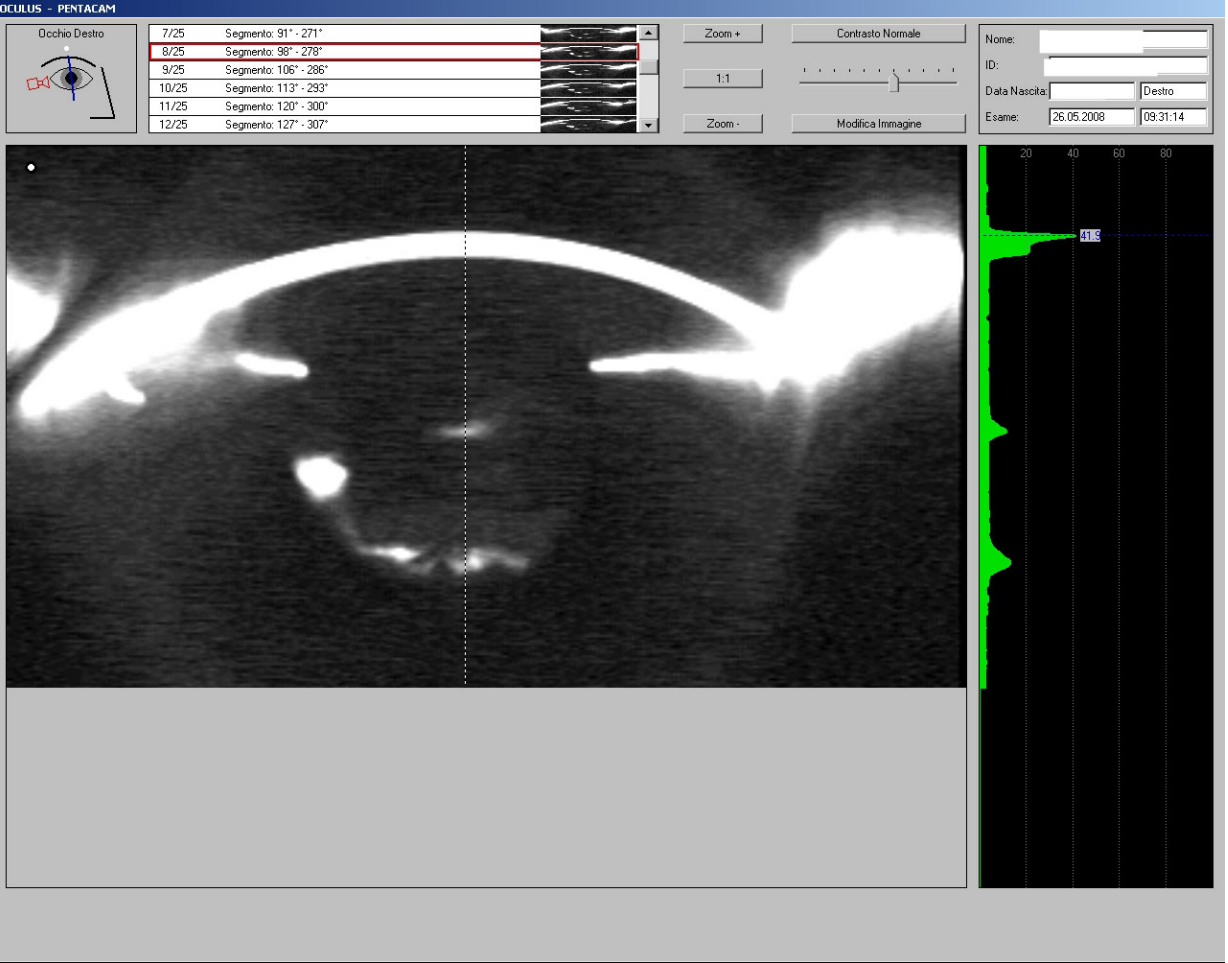
**Pentacam image showing the presence of slightly milky fluid behind the IOL and a distended capsular bag**.

The patient was placed on hypotonizing topical therapy, and a surgical peeling of the anterior capsule was performed. The following days the slit lamp examination showed the presence of slightly milky fluid behind the IOL and a distended capsular bag, that was confirmed by Pentacam (Fig. 2), that were not detected before for the presence of the opaque anterior capsule. The IOP was still 30 mmHg. A YAG laser posterior capsulotomy was attempted but no decrease in the IOP was obtained.

## Discussion

In the international literature we found several cases of ACP or CBS, but we were unable to find an association of these two complications.

ACP depends on contraction of a fibrous membrane that can be present either beneath the anterior capsule or on the outer surface of the anterior capsule [6].

Although the pathogenic mechanisms responsible for excessive capsule fibrosis and contracture are not well understood, several histopathologic studies have identified the cell types associated with pseudophakic fibrosis [2].

Some studies suggest that the IOL optic material may influence the development of anterior capsule fibrosis. Intraocular lens shape, IOL biomaterial, and, more specifically, the hydrophilicity of the biometarial have been associated with a high degree of postoperative inflammation [7,8].

It has been suggested that surgical invasion and contact with the IOL stimulate residual lens epithelial cells (LECs) to produce cytokines. These cytokines may induce collagen production and fibrous proliferation.

Some of the cytokines induce transformation of the LECs into myofibroblasts. that contain contractile filaments [9].

In parallel with these processes, aqueous prostaglandin E2 concentration is elevated, leading to blood aqueous barrier breakdown and an increased aqueous protein concentration [10].

Previous studies have demonstrated disruption of both the blood-retinal and blood-aqueous barriers in eyes of patients with retinitis pigmentosa [4]. An increase in blood-derived cytokines in the aqueous humor of these eyes after cataract surgery, which results in increased activation of LECs with fibrosis and contracture of the anterior capsule.

The same mechanism could be present in patients with Steinert Distrophy, but in our patient we believe that the mechanism of cytokine production was induced by the unnoticed CBS. This is supported by Sorenson [6] who reported that in patients with CBS the observation resulted in acute angle closure glaucoma, chronic anterior chamber inflammation, and development of posterior capsular opacification.

In limited forms of capsular shrinkage without invasion of the optical zone, YAG laser capsulotomy is considered the first choice. In severe cases with dense fibrous plaques, laser capsulotomy will result in incompletely resolved capsular debris, enhancing the risk for inflammation and recurrence [9]. For this reason, and to avoid the risk of further rise in the IOP due to these debris, we decided to perform the surgical approach to take off the fibrotic tissue.

In case of CBS the prompt treatment should restore the IOP. Unfortunately in our patient we did not succeed; the reason for this could have been the late posterior capsulotomy treatment due to the absence of the patient at the follow up visit, that leaded to inflammation and fibrosis of the capsular bag. In conclusion, our case report could suggest to perform further studies to check if the implantation of hydrophilic lenses should be avoided in myotonic patients and to closely follow patients after cataract surgery because in case of CBS development a prompt posterior capsulotomy should be performed to avoid more severe complications.

## Competing interests

The authors declare that they have no competing interests.

## Consent

Written informed consent was obtained from the patient for publication of this case report and accompanying images. A copy of the written consent is available for review by the Editor-in-Chief of this journal.

## Authors' contributions

NR has made substantial contributions to conception and design, analysis and interpretation of data; he has given final approval of the version to be published. ML has been involved in drafting the manuscript, performed the photographic description of the case. MDB has been involved in the research of the bibliographic data. MB has been involved in drafting the manuscript. LP played an important role in the selection of the patient, has given final approval of the version to be published. All authors read and approved the final manuscript.
